# Shaping the Female Microbiome: A Review of Lifestyle Factors Influencing the Vaginal, Gut, Oral, and Skin Microenvironments

**DOI:** 10.1007/s00248-026-02747-w

**Published:** 2026-03-29

**Authors:** Isabella M. Davidson, Elham Nikbakht, Hayley M. O’Neill, Larisa M. Haupt, Paul Joseph Dunn

**Affiliations:** 1 Faculty of Health Science & Medicine, Bond University, Gold Coast, Robina, Australia; 2 Stem Cell and Neurogenesis Group, Genomics Research Centre, Centre for Genomics and Personalised Health, School of Biomedical Sciences, Queensland University of Technology (QUT), 60 Musk Ave., Kelvin Grove, Brisbane, Queensland 4059 Australia; 3 ARC Training Centre for Cell and Tissue Engineering Technologies, Queensland University of Technology (QUT), Brisbane, Australia; 4 Max Planck Queensland Centre for the Materials Sciences of Extracellular Matrices, Queensland University of Technology (QUT), Brisbane, Australia

**Keywords:** Female microbiome, Modifiable factors, Women’s health, Microbial diversity

## Abstract

The female microbiome, spanning the vaginal, gut, oral, and skin sites, harbours distinct microbial communities. Although the diversity and function of microbial communities across these sites are becoming increasingly understood, the extent to which modifiable lifestyle and environmental factors such as smoking, diet, alcohol intake, obesity, physical activity, stress, hygiene, and sexual behaviours shape these microbiomes remains underexplored. This review is restricted to modifiable lifestyle and environmental factors and does not comprehensively assess pharmaceutical exposures (e.g., antibiotics or hormonal therapies) or hormonal influence. To date, no review has comprehensively assessed and compiled evidence across the four microbial sites in females, despite their unique hormonal, physiological, and reproductive characteristics that distinctly influence microbial composition and function. This review provides a comprehensive examination of how such factors influence the dynamics of microbial composition and function along with site-specificity while also assessing cross-site microbial interactions. We focus exclusively on females to address a critical knowledge gap to provide a foundation from which future research and interventions can be tailored to women’s health. This review discusses the underlying mechanisms driving microbial shifts and their impact on host health, highlighting critical gaps in our current knowledge. The integration of findings from multi-site microbiome research, highlights the potential to inform targeted, preventative, and therapeutic strategies that utilise the inherent dynamic nature of the microbiome to improve health outcomes across the female lifespan.

## Introduction

The human microbiome plays a critical role in the regulation of immune responses, metabolic processes, and overall health, with distinct differences observed between males and females. These differences arise largely due to variations in sex hormones, body composition, and reproductive physiology, which shape microbial communities across multiple body sites [1, 2]. In females, cyclical hormonal changes, pregnancy, and reproductive events uniquely impact microbial communities, leading to differing patterns of diversity and microbial composition to those in males [3]. These factors create unique microbial niches—most notably within the vaginal, gut, oral, and skin microbiomes—each with distinct microbial abundance and clinical relevance [4]. Female sex hormones are key determinants that directly shape and mediate the influence of external exposures on the microbiome. Hormonal fluctuations across the menstrual cycle and pregnancy are significant drivers of microbial landscapes often resulting in shifts in microbial communities [5, 6]. Fluctuations in oestrogen and progesterone throughout the menstrual cycle, result in differences in microbial stability, diversity, and resident microbe present [7]. Medication exposures including antibiotics and hormone-modulating therapies are also recognised as potent modifiers of microbial landscapes through reductions in diversity, introduction of beneficial microbiomes, or providing metabolites for microbes [4, 7–9]. However, due to the large variety of pharmaceutical treatments that may influence microbiome composition, and previous reviews in this space, a comprehensive review of pharmacologic influence on the female microbiome was deemed beyond the scope of this review [10–14].

While these female-specific biological drivers and medication exposures provide important context, this review focuses on modifiable, non-pharmacologic lifestyle and environmental factors. Despite sex-specific differences, most research either combines male and female data, or focuses solely on a singular body site limiting our understanding of how different microbial sites interact and contribute to female health. Understanding these sex-specific differences is particularly important given the influence of the female microbiome to reproductive health and disease. As an example, the female genital tract microbiome, encompassing the vagina, cervix, uterus, and endometrium, has been shown to directly impact fertility and overall reproductive outcomes [15–18].

Microbial imbalance, or dysbiosis, refers to a microbial imbalance that negatively influences the human body due to the loss or gain of certain microbes and their relative abundance [19]. Emerging evidence has highlighted strong associations between dysbiosis and a range of adverse health outcomes, including human immunodeficiency virus (HIV), pelvic inflammatory disease (PID), sexually transmitted infections (STIs), urinary tract infections (UTIs), endometriosis, miscarriage, preterm birth, metabolic and cardiovascular disorders, hormone dysregulation, acne, bacterial vaginosis (BV), gingivitis, dental caries, and inflammatory skin diseases [1, 2, 20–22]. Notably, microbial compositions vary not only across different anatomical sites, but also between different individuals, further underpinning the variability and complexity of the female microbiome [2].

Due to the potential for future therapeutic interventions, there is a growing interest in understanding how modifiable lifestyle and environmental factors shape the female microbiome. These factors represent promising intervention targets for disease prevention, early detection, and therapeutic interventions [23]. However, current research is limited by its narrow scope, often focusing on only one or two microbial sites, with the vaginal and gut microbiomes the most common studies, failing to comprehensively assess the broad range of modifiable factors influencing microbial composition and function. To date, no review has collated evidence across the four major microbial sites in females, despite the interconnection of each site is and the impact on female health. This review therefore addresses a critical knowledge gap by focusing exclusively on females and synthesising evidence across the vaginal, gut, oral, and skin microbiome sites. The identification of the most influential factors in this review aims to provide the foundation for future female-focused research and to inform targeted strategies for prevention and treatment of a range of disorders.

**Fig. 1:**
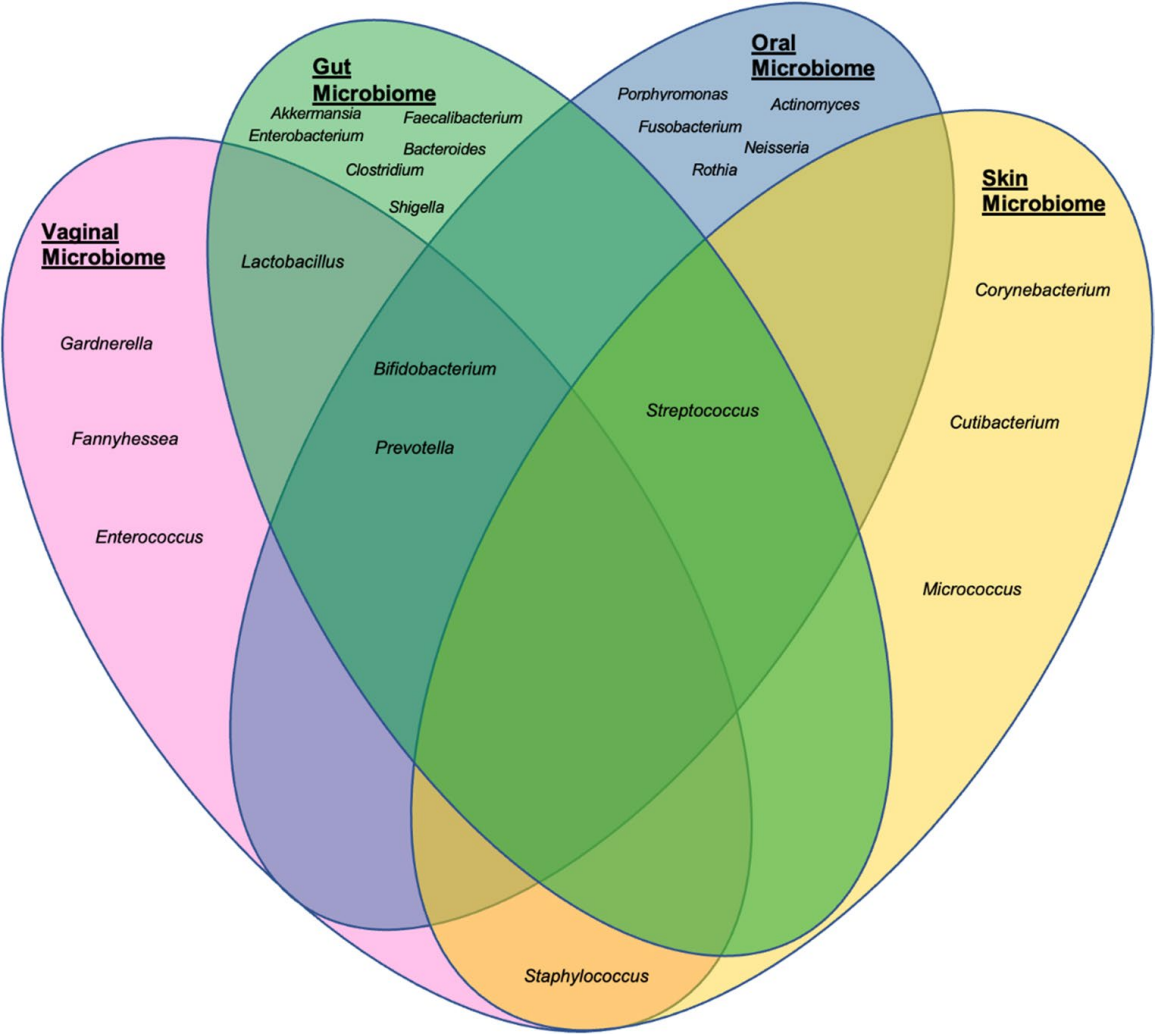
Summarisation of microbial overlap in vaginal, gut, oral, and skin sites for females illustrating unique and shared taxa

Microbial communities differ by anatomical site, however, subsets of microbial species are often found across multiple anatomical sites highlighting the potential for shared exposures and mechanisms to shape multiple microbial populations at once (Fig. 1) [1, 4, 15, 23]. For example, microbes such as *Bifidobacterium* and *Prevotella* are commonly found in the vagina, gut, and oral sites whereas *Streptococcus* can be found in the gut, oral and skin microbiomes and therefore alterations in one of these microbes may result in alterations in all of its inhabited microbial sites [4, 15, 24].

**Fig. 2:**
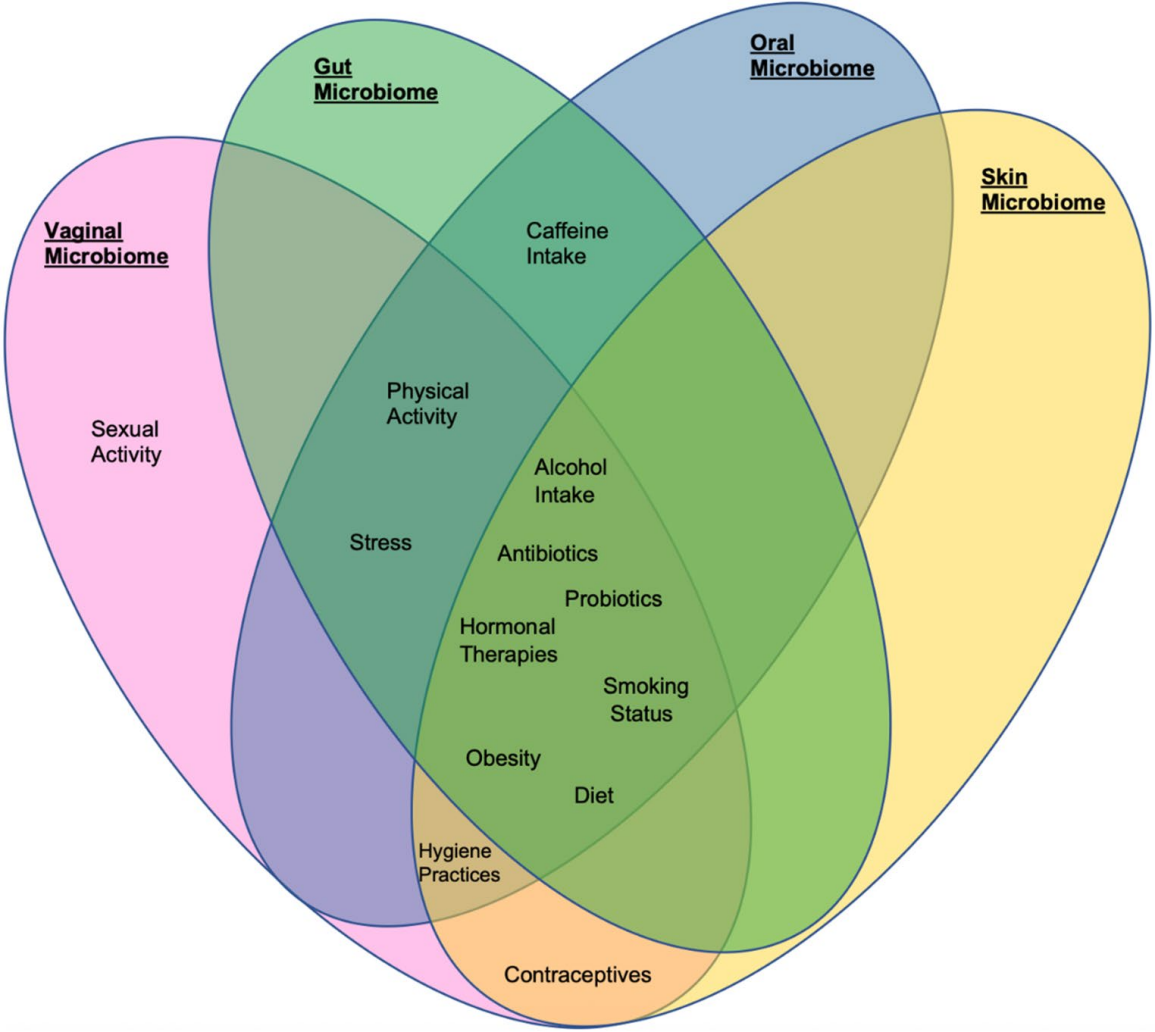
Schematic representation of lifestyle factors and their influence on the female microbiome across anatomical sites. Factors influencing one or more microbial sites are indicated by overlapping regions, illustrating shared influences across sites. Microbiome sites are colour-coded as follows: vaginal (pink), gut (green), oral (blue), and skin (yellow). The diagram highlights both site-specific and cross-site effects of factors such as sexual activity, stress, physical activity, caffeine intake, alcohol intake, obesity, smoking status, diet, hygiene practices, hormonal therapies, antibiotics, probiotics, and contraceptive use on microbial composition

This work explores the impact of lifestyle and environmental factors for their influence on microbial diversity and composition in females across the vaginal, gut, oral, and skin microbiomes, which exhibit distinct associations with microbial α- and β-diversity and specific microbes () [7, 25–27]. For instance, diet-related factors such as macronutrient intake, dietary patterns, and alcohol consumption often drive changes in microbial diversity and the abundance of particular bacterial groups, while stress, smoking, and sexual behaviours have been found to be commonly linked to dysbiosis and the reduction of protective species such as *Lactobacillus* [7]. In contrast, physical activity, and certain dietary patterns, have been frequently associated with higher microbial diversity and beneficial microbial profiles [7, 25, 28]. The influence of each factor on the composition and dynamics of the respective microbiomes illustrated in Fig. 2 with a summary of these associations across the distinct anatomical sites, outlining both beneficial and negative microbial alteration associated with lifestyle factors, summarised in Table 1.

**Table 1:**
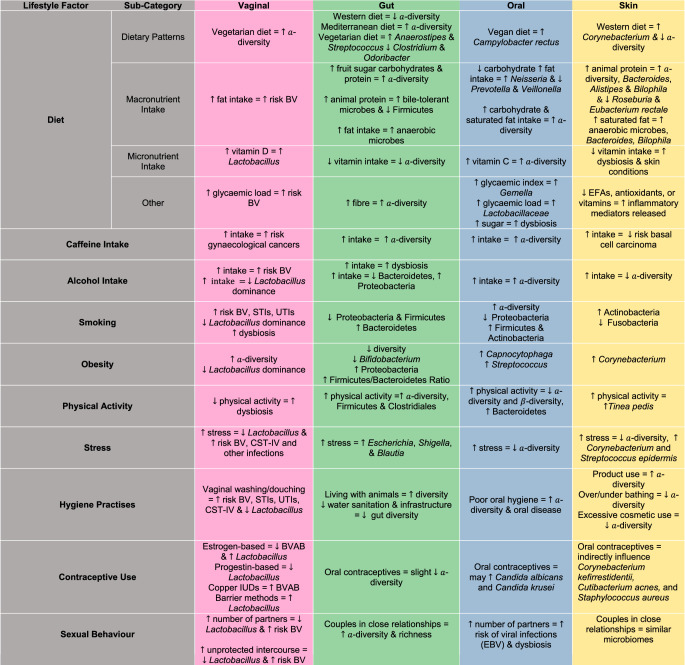
Overview of lifestyle factors including diet, smoking, alcohol intake, obesity, physical activity, stress, hygiene practices, caffeine intake, contraceptive use, and sexual behaviour organised by their overall impact and extent of influence across different microbiome sites which include the vaginal (pink), gut (green), oral (blue), and skin (yellow) microbiomes which are colour coded to reflect anatomical relevance. The table outlines associations with microbial diversity (α- and β-diversity) and specific microbes, highlighting whether the factors are linked to beneficial or adverse microbial shifts

Abbreviations: *BV* bacterial vaginosis, *STIs* sexually transmitted infections, *UTIs* urinary tract infections, *CST* community state type, *BVAB* bacterial vaginosis associated bacteria, *EBV* Epstein-Barr Virus, *EFA* essential fatty acid

## Dietary Habits

**Fig. 3:**
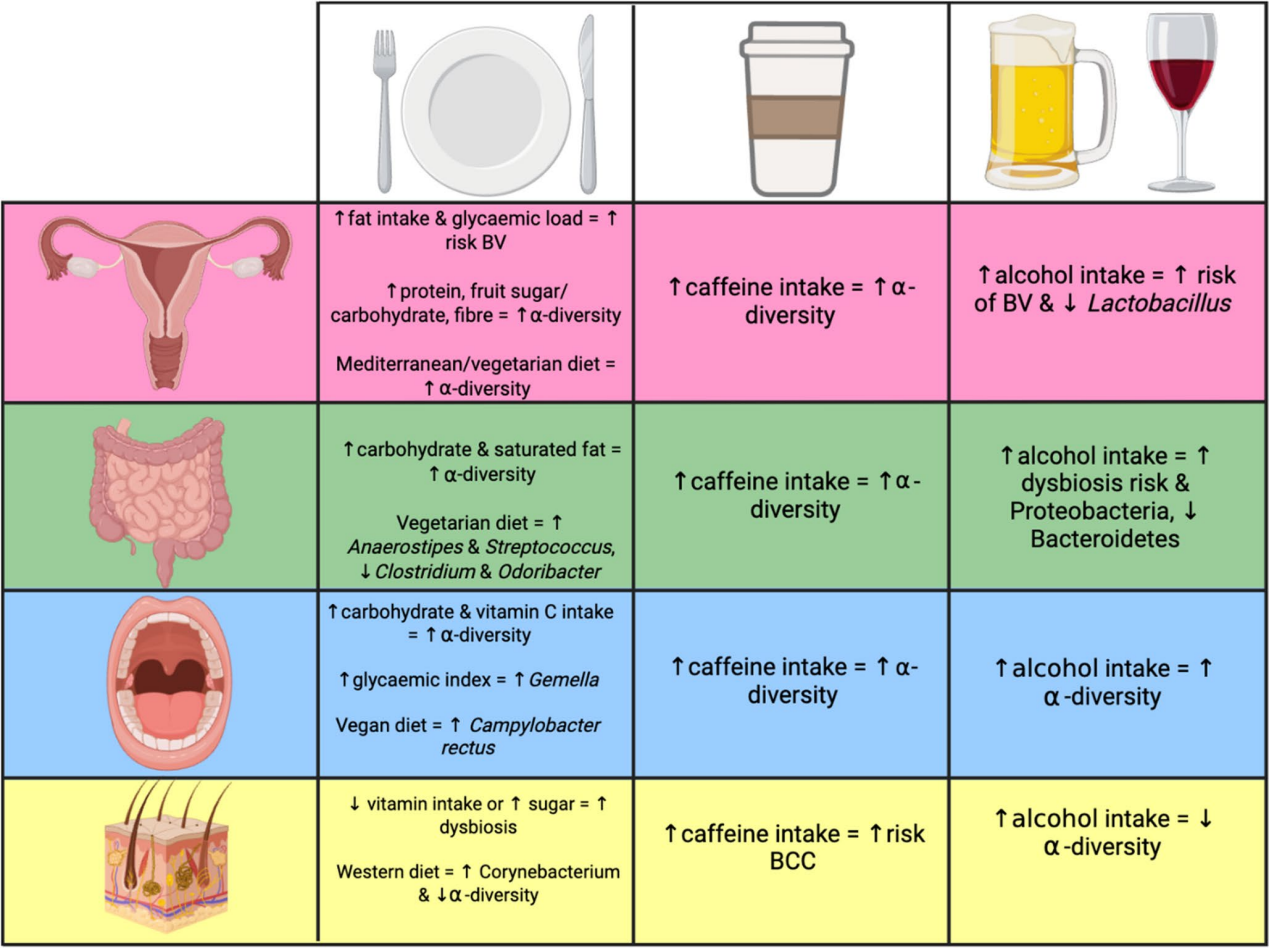
Schematic representation of the influence of dietary habits including caffeine and alcohol intake on the vaginal (pink/first row), gut (green/second row), oral (blue/third row), and skin (yellow/fourth row) microbiomes. Dietary factors include overall dietary patterns (Western, Mediterranean), macronutrient composition (carbohydrates, proteins, fats), and micronutrient intake (vitamins, minerals), all of which influence microbial diversity and composition. Image created in BioRender. Abbreviations: BV, bacterial vaginosis; BCC, basal cell carcinoma

The impact of dietary habits including nutrient intake, caffeine, and alcohol use on the gut microbiome has been extensively studied, with strong associations identified between dietary intake and microbial composition, whereas the relationships between the vaginal, oral, and skin microbiomes remain less well explored (Fig. 3) [29–31]. Diet can influence microbiome structure and function by providing substrates to promote or inhibit the growth of specific microbial communities, altering local environmental conditions such as pH and nutrient availability, and modulating host immune and metabolic responses [25, 32–34]. Caffeine and alcohol intake can also influence microbiome balance. Caffeine has been linked to changes in microbial diversity and potential protective effects, while alcohol has generally been associated with dysbiosis and disruption to microbial composition. Although less studied than diet, these factors may similarly shape microbiome dynamics to drive shifts in microbial composition across multiple body sites, contributing to health and disease risk (Fig. 3).

### Vaginal Microbiome

Diet has an important role in shaping the composition and diversity of the vaginal microbiome through direct and indirect mechanisms. Dietary composition can indirectly influence the vaginal microbiome through the gut-vagina axis as the gut can indirectly influence the vagina through short-chain fatty acid (SCFA) productions, changes in oestrogen metabolism, and microbial translocation from the gut to vagina [4]. Microbial imbalance in the gut due to diet, can result in reduced protective SCFA release which may induce gut inflammation leading to systemic inflammation and influencing the vaginal environment through inflammatory mediators [4]. Additionally, dietary components which negatively alter the gut microbiome, can result in changes in oestrogen metabolism such as reduced oestrogen which can reduce vaginal *Lactobacillus* and increase vaginal dysbiosis risk [8, 35].

Dietary composition directly influences microbial communities through overall dietary patterns rather than individual nutrients with plant-based diets linked to increased diversity when compared with meat-based diets [7]. For example, women who consume a vegetarian diet exhibited increased $\:\alpha\:$-diversity in comparison with non-vegetarians [7, 28]. Diets rich in fibre and starch have also been associated with favourable vaginal microbiome profiles and reduced risk of BV [36, 37]. Specifically, an increased intake in dietary fibre has been significantly linked to lower concentrations of female reproductive hormones such as oestrogen, progesterone, follicle stimulating hormone (FSH), and luteinising hormone (LH). While most mechanistic aspects remain unknown, a reduction in oestrogen due to high fibre can be due to a reduction in oestrogen reabsorption in the gut and binding to oestrogen in the intestine, increasing excretion [38]. Similarly, a diet comprised of complex carbohydrates—assessed by glycaemic index (GI), glycaemic load (GL), healthy eating index (HEI) and Nordic nutrition recommendations (NNR)—has been linked to reduced BV risk and vaginal microbiome stability [39].

In contrast, certain dietary patterns promote dysbiosis and shift the microbiome towards BV-associated states. A diet deficient in vitamin D has been found to be associated with higher relative abundances of potentially pathogenic microbes such as *Megasphaera* [7]. Diets dominated by high fats—including high saturated fats and glycaemic load—have been shown to be associated with an increased risk of developing BV and severe BV through accumulation of bacterial vaginosis associated bacteria (BVAB) [7, 23, 36, 40]. Other studies have suggested increased saturated fat intake independent of energy intake as a predictor for BV and BVAB [40], with diets deficient in micronutrients (including vitamins A, C and E, $\:\beta\:$-carotene, folate, betaine, calcium and zinc) have also been identified to be negatively associated with vaginal health homeostasis and to increase the risk for BV and infections such as *Human Papilloma Virus (HPV)* [23, 36, 41]. Interestingly, the relationship between caffeine intake and the vaginal microbiome remains limited, with research to date focusing on intake and gynaecological cancers rather than microbial changes [42]. Additionally, increased intake of alcohol has been associated with many gynaecological cancers [42], and suggested to increase vaginal $\:\alpha\:$-diversity and to simultaneously decrease the abundance of resident genera *Lactobacillus* resulting in an increased risk of developing BV [43].

### Gut Microbiome

Diet significantly impacts the resident microbes within the female gut with dietary patterns and nutrient intake identified to be associated with alterations in relative microbial abundance throughout the gut [44]. Data to date suggests diets high in fibre to be recommended with non-digestible fibres serving as prebiotics that can stimulate beneficial gut microbes, supporting the production of beneficial metabolites and increase of overall strain diversity [45, 46]. For females, inclusion of dietary probiotics have also been linked with improved gut barrier integrity, pathogen resistance, and reduced inflammation via the introduction of beneficial microbes [4, 23]. Consumption of a western style diet—characterised by high fat, animal protein, simple sugars, and low dietary fibre—has been found to reduce $\:a$-diversity and microbes such as *Bifidobacterium* and *Eubacterium* whilst increasing inflammatory bacterial profiles via an increased Firmicutes/Bacteroidetes ratio [47]. The western diet has also been strongly associated with an increased risk for chronic disease development due to its poor nutritional quality [25]. The Mediterranean diet—characterised by plant-based foods, and healthy fats— results in reduced *Bifidobacterium* but increased $\:a$-diversity, along with *Prevotella*, and *Ruminococcaceae* abundances, both associated with weight loss. A vegetarian diet showed an increased abundance of *Anaerostipes* and *Streptococcus* in conjunction with reduced abundance of *Clostridium* and *Odoribacter* [47]. Interestingly, a rural diet has been shown to significantly alter the gut microbiome through an increased abundance of Bacteroidetes and reduction in Firmicutes along with a significantly higher production of SCFAs. Comparative analysis identified the gut microbiota associated with a rural diet to supports greater energy extraction from fibre and promotion of an anti-inflammatory response via increased SCFA production [48].

A diet high in carbohydrates sourced from fruit-derived sugars was identified to promote an increase in abundance of *Bifidobacterium* and a reduction in *Bacteroides*, thereby increasing $\:a$-diversity [44, 47]. Protein consumption also alters microbial composition with the source of protein also linked with total microbial diversity in the gut. As an example, a high intake of animal protein has been associated with increased *Bacteroides fragilis* and *Bacteroides vulgatus* when compared to an animal protein-free diet [47]. In contrast, protein intake from non-animal sources, such as pea powder protein, has been shown to increase the relative abundance of *Bifidobacterium* and *Lactobacillus*, whilst also promoting SCFA production, anti-inflammatory properties, and intestinal barrier integrity [47, 49]. Diets rich in animal proteins have also been identified to increase bile-tolerant microbes (such as *Alistipes*, *Bilophila*, and *Bacteroides*) and reduce Firmicutes that metabolise plant polysaccharides (such as *Roseburia*, *Eubacterium rectale*, and *Ruminococcus bromii*), with a diet high in plant consumption found to result in an inverse profile [49]. A diet high in fats has been shown to significantly alter the gut microbiome through the increased abundance of anaerobic bacteria and microbes such as *Bacteroides* resulting in increased susceptibility for bacterial translocation within and outside of the gut [47]. Assessment of a micronutrient low diet, such as vitamins B, C, D, and E along with minerals such as calcium, magnesium, zinc and iron, has been identified to be associated with reduced $\:a$-diversity, impaired microbial function and an increased gut inflammatory profile [33]. A diet of high dietary soluble fibres has been shown to increase $\:a$-diversity, *Bifidobacterium*, and *Lactobacillus* in the gut while inhibiting inflammation, leaky gut, DNA damage and cancer progression through carbohydrate fermentation and SCFA production from plant-based foods [46, 47].

Additionally, caffeine intake has been identified as a large influencer of the gut microbiome where coffee drinkers exhibit a distinguishable environment compared to non-coffee drinkers. The abundance of *Leptotrichia asaccharolytica* has shown the strongest association to coffee consumption as well as increased caffeine intake associated with an increased $\:\alpha\:$-diversity and higher relative abundance of *Faecalibacterium* and *Alistipes* whilst having a lower abundance of *Erysipelatoclostridium* [50, 51]. Alcohol consumption also demonstrates significant influences on the gut microbiome with an increased intake of alcohol associated with reduced Bacteroidetes and higher abundances of Proteobacteria and Fusobacteria [52]. Increased alcohol consumption linked with gut dysbiosis and systemic inflammation which can lead to downstream health implications such as inflammatory bowel disease (IBD), heart disease and much more [53].

### Oral Microbiome

Diet can also influence the oral microbiome, with particular dietary patterns found to promote beneficial shifts and others to disrupt microbial balance. As an example, vegetarianism is linked to significantly altered oral microbiota compositions compared with omnivores in both microbial diversity and community population representation [54]. Vegetarianism has been linked to an increase in anti-inflammatory mediators such as interleukin-10 and microbes associated with periodontal stability *Rothia* and *Peptidiphaga sp. HMT183* [55]. Individuals following a vegan diet, have also been found to have significantly increased levels of *Campylobacter rectus* in comparison to those following an omnivorous diet [54]. Additionally, a Mediterranean diet has shown to reduce the abundance of microbes associated with periodontal disease such as *Treponema denticola*, *Prevotella intermedia*, and *Porphyromonas gingivalis* and increase beneficial microbes such as *Streptococcus cristatus* [55]. Diets comprising of anti-inflammatory components such as fruits, vegetables, and legumes are linked to a healthy oral microbiome comprised of beneficial microbes including Firmicutes, Proteobacteria, Actinobacteria, Bacteroidetes, Fusobacteria, and Spirochaetes whereas diets comprised mainly of inflammatory food including high sugar and high saturated fats contributes significantly to a dysbiotic oral microbiome leading to growth of pathogenic organisms such as *Streptococcus mutans* and *sobrinus*, *P gingivalis*, and *Tannerella forsythia* [32, 55].

The most significant impact on oral microbiome composition has been identified to be high sugar intake, with sugar-rich diets significantly influencing oral dysbiosis through enrichment of microbes such as *Streptococcus*, *Scardovia*, *Veillonella*, *Rothia*, *Actinomyces*, and *Lactobacillus* [32]. A diet low in carbohydrates but high in fat has also been shown to impact the oral microbiome through an increased relative abundance of *Neisseria* and a reduction in abundance of microbes such as *Prevotella* and *Veillonella* often associated with improved oral health [34]. High carbohydrate intake has been associated with increased $\:a$-diversity and an increased abundance of *Fusobacteria* and *Leptotrichia* whilst also being associated with a reduced abundance of *Actinomyces* [56]. Interestingly, a diet high in saturated fats has been associated with an increased $\:a$-diversity and increased abundance of Betaproteobacteria and Fusobacteriota. Increased glycaemic index has also been identified to be associated with microbial composition changes in the oral microbiome through increased *Gemella*, with increased glycaemic load associated with an increase in *Lactobacillaceae* abundance [56, 57]. An increased intake of dietary fibre has also shown to increase the abundance of both *Capnocytophaga* and *Neisseria subflava* [54]. Additionally, micronutrient intake was found to influence oral microbial abundance, with vitamin C specifically identified to increase $\:a$-diversity and Fusobacteriota, *Leptotrichiaceae* and *Lachnospuraceae* abundances [57].

In addition, intake of caffeinated beverages such as tea has also been shown to directly influence the oral microbiome with increased intake associated with increased $\:\alpha\:$-diversity and lower relative abundance of *Bifidobacteriaceae*, and Lactobacillales and higher relative abundance of Fusobacteriales, and Clostridiales. In contrast, coffee consumption was not found to be associated with any significant microbiological changes within the oral cavity in the same cohort of NCI PLCO and ACS CPS-II participants which included females from the United States (*n* = 938) [58]. Interestingly, another study recorded a statistically significant increase in $\:\alpha\:$-diversity and $\:\beta\:$-diversity in coffee drinkers when compared with non-coffee drinkers [59]. Alcohol consumption has also been found to significantly impact the oral cavity with increased alcohol consumption associated with reduced abundance of Lactobacillales and increased $\:\alpha\:$-diversity and an increased abundance of Proteobacteria and Actinobacteria were increased [60].

### Skin Microbiome

The indirect impact of diet on the skin has been regularly researched alongside the gut microbiome where direct dietary impacts on the skin microbiome are significant in lipid regulation. Whilst the skin and gut microbiomes are not connected anatomically, gut dysbiosis has shown to result in negative skin manifestations due to increased inflammation which can result in skin lesions and disturbed skin microbes [2, 61]. A diet that dysregulates gut barrier integrity results in an increase in inflammatory mediator and metabolite release which can indirectly result in skin inflammation. Increased inflammation can result in inflammatory skin conditions such as psoriasis and alter microbial signatures depending on inflammatory mediators released [2, 61, 62]. Dysregulated gut barrier integrity also allows microbes to enter the bloodstream which in combination with inflammatory mediators, may interact with skin receptors, directly influencing resident skin microbiota [63]. A deficiency in essential fatty acids (EFAs), antioxidants, or vitamins in diet may influence the synthesis and metabolism of skin lipids ultimately impacting skin barrier integrity Diets high in fruits, vegetables, fibre, omega-3 and 6 fatty acids, and other EFAs, are linked to significant enhancements in skin hydration, barrier function, and reduced inflammation. Conversely, increased intake of saturated fats have been linked to increased skin inflammation, directly altering resident microbes and leading to manifestations including acne vulgaris [64].

Dietary patterns can also directly influence skin microbial composition with the western diet identified to be associated with increased relative abundance of *Corynebacterium* and decreased $\:a$-diversity [65]. Diets rich in carbohydrates (both digestible and undigestible), proteins, and fats, alter host immunologic and metabolic profiles, resulting in altered microbial composition [66, 67]. The consumption of animal-based proteins has been demonstrated to increase the relative abundance of microbes such as *Bacteroides*,* Alistipes*,* Bilophila* and overall $\:a$-diversity of the skin. Additionally, diets high in saturated fat have been associated with an increased *Bacteroides* and *Bilophila* along with an overall increase in anaerobes [67]. Diets lacking vitamins A, B3, C, D, and E micronutrients have all shown increased susceptibility to skin dysbiosis through increased colonisation of pathogenic microbes such as *Staphylococcus aureus*, *Cutibacterium*, and *Aspergillus*. The increased colonisation of pathogenic microbes results in gut-skin axis impairment and released cytokines resulting in downstream inflammation and skin manifestations [66].

Currently, there is little information regarding the influence of caffeine intake on skin microbiota with findings suggesting increased or moderate caffeine intake to be associated with decreased basal cell carcinoma (BCC) risk due to the anti-carcinogenic and antioxidant capacity of caffeine [68]. Research is also scarce regarding alcohol intake and the skin microbiome however, the microbial diversity of the skin in alcohol drinkers has been found to be significantly different to non-alcohol consumption resulting in increased microbial diversity over drinkers [59]. Additionally, alcohol consumption has been shown to result in skin barrier disturbances and lead to different skin infections [69].

Overall, a range of dietary factors such as diet, caffeine, and alcohol consumption can significantly shape microbial abundance and diversity across multiple body sites. Different macro- and micronutrients have been found to support microbial balance or drive dysbiosis within the female body, with caffeine intake linked to shifts in microbial diversity. Similarly, alcohol consumption can influence microbial composition across the vaginal, gut, oral, and skin microbiomes, with further research needed to more fully understand the mechanisms mediating these affects along with the range of implications of these changes on women’s health.

## Smoking Status

Smoking has been shown to significantly influence microbial composition across the body, disrupting the balance between protective and pathogenic species. These changes can drive dysbiosis and inflammation through mechanisms such as the introduction of harmful metabolites, suppression of beneficial microbes, and modulation of immune and hormonal pathways [7, 23, 70]. The extent of these effects can be dose-dependent, with heavier smoking found to be associated with greater microbial disruption [71, 72]. Overall, smoking has been linked to alterations in sex hormone production which have included elevated follicular phase steroid metabolites, reduced progesterone in the luteal phase, and overall shorter cycle lengths [73].

### Vaginal Microbiome

In females that smoke, *Lactobacillus spp*. abundance in the vagina has been identified to be significantly reduced with levels of harmful metabolites and hormonal alterations which increase the risk of vaginal infections such as HPV, STIs, and BV found to be increased [74]. Smoking has been associated with an altered hormonal profile including reduced luteal and mid-cycle oestradiol levels exhibiting an anti-oestrogenic effect [70, 75]. Increased smoking prevalence has been associated with increased BV incidence, higher likelihood of CST-IV classification, lower *Lactobacillus spp.* abundance, and an increase in metabolites associated with inflammation [7, 23, 36, 76, 77]. Smoking has been demonstrated to impact the vaginal microbiome in a dose-dependent manner through an anti-estrogenic influence that negatively influences *Lactobacillus* abundance [70, 71].

### Gut Microbiome

Interestingly, smoking has also been linked with gut health and the gut microbiome with smokers demonstrated to have an increased abundance of *Bacteroides* along with changes in microbial populations potentially contributing to an increased risk of inflammatory bowel diseases (IBDs) such as Crohn’s disease [25]. Smoking has been demonstrated to significantly alter the gut microbiome influencing overall community composition, and microbial metabolites produced. Smoking has also been suggested as an inhibitor of oestrogen synthesis resulting from inhibition by tobacco alkaloids and nicotine [78]. Additionally, with smoking acting as an antimicrobial agent, the relative abundance of Firmicutes and Proteobacteria has been found to be reduced in smokers and the phylum Bacteroidetes to be increased when compared with non-smokers [79].

### Oral Microbiome

The oral microbiome in smokers has been shown to be different on all taxonomic levels and to exhibit a highly diverse, anaerobic, and pathogenic rich environment reflective of a disease-associated community in comparison to non-smokers. The oxygen depleted environment produced from smoking is suggested to influence a shift in microbial communities in the oral cavity to anaerobes such as *Veillonella* and *Actinomyces* [80]. Smoking has been suggested to increase the risk of oral biofilms and dental caries due to the increase of known pathogens such as *Filifactor alocis* [81]. In smokers, the abundance of Proteobacteria has also been found to be reduced with increased Firmicutes and Actinobacteria contributing to a shift towards dysbiosis and increased inflammation within the mouth [82, 83].

### Skin Microbiome

Whilst data continues to emerge, smoking has been found to have an impact on skin resident microbes, with an elevated level of Actinobacteria observed in smokers along with reduced abundance of Fusobacteria when compared to healthy controls. In addition, significant differences in $\:\beta\:$-diversity between non-smokers, light smokers, heavy smokers has been identified, with a large number of microbes altered in relation to smoking status [72].

Overall, smoking has been identified to exert widespread effects on microbial communities across multiple body sites, driving dysbiosis and increasing susceptibility to infection and inflammation. These findings highlight the importance of smoking cessation as a potential strategy to restore microbial balance and improve female health outcomes.

## Obesity

Obesity is a rapidly escalating global health concern, impacting approximately 1 in 8 individuals worldwide, with a higher prevalence in females [84]. Obesity can occur across the female lifespan, with rising rates observed in children, adolescents, and adults due to a wide range of mechanisms [84, 85]. Childhood obesity has been linked to an earlier age of menarche, causing a surge in female pubertal hormones, and earlier age of onset of breast development then girls at a healthy weight [86]. The shifts in growth maturation and sex-hormones occurring from obesity along with global dietary patterns shifting toward processed foods, saturated fats, and refined sugars, alongside reduced intake of dietary fibre has been postulated to influence downstream microbiome development [25, 87].

**Fig. 4:**
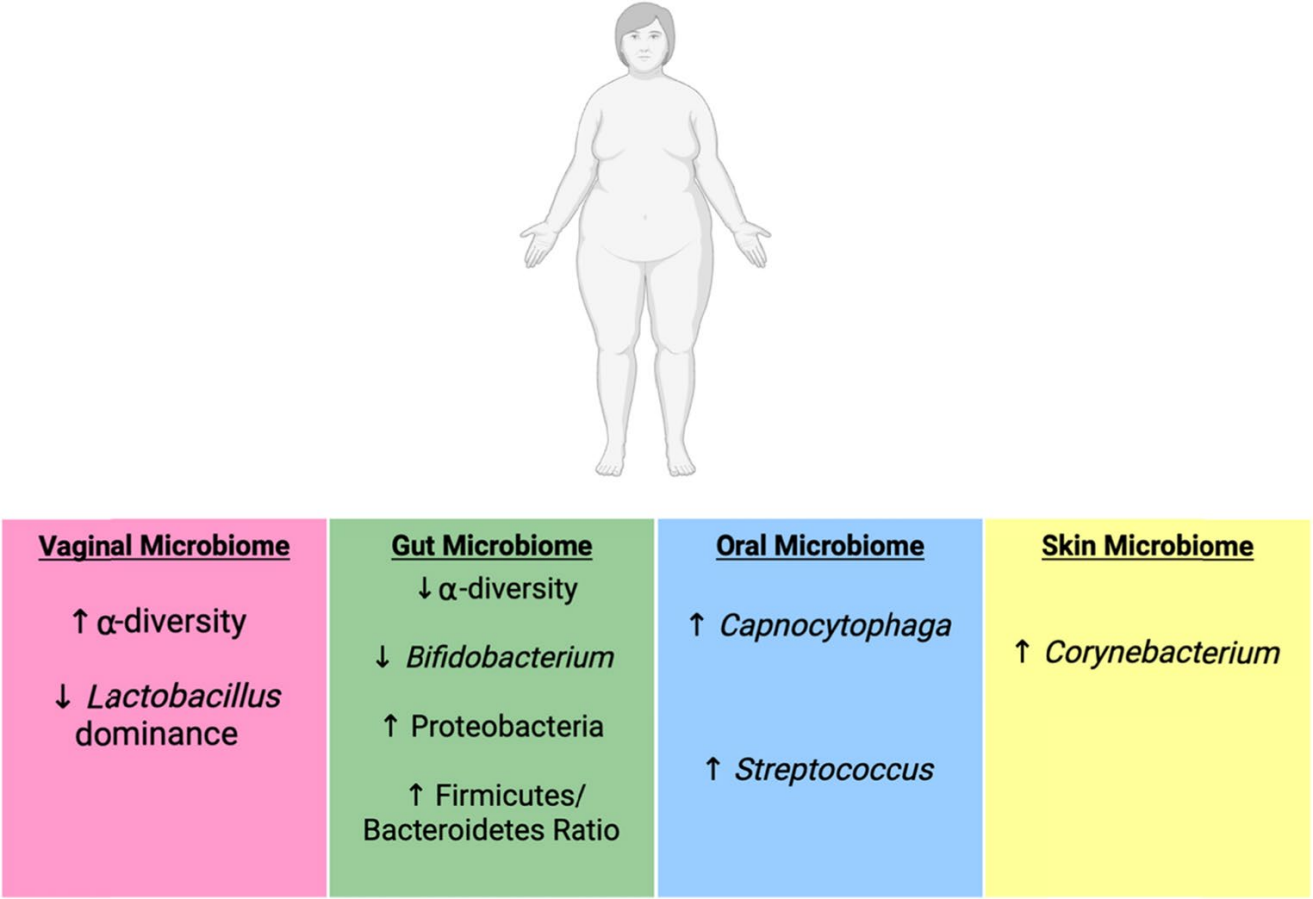
Representation of the influence of overweight/obesity on the composition and diversity of the female microbiome across four anatomical sites. Microbiome sites are colour-coded for clarity: vaginal (pink), gut (green), oral (blue), and skin (yellow). Arrows indicate the direction and nature of microbial change, with reductions in diversity and beneficial microbes contrasted with an increase in potentially pathogenic microbes. Image created in BioRender

Obesity can be defined as an excessive increase in body fat that may increase health risks, characterised by a body mass index (BMI) $\:\ge\:$ 30 [85]. Microbial changes associated with overweight/obesity have been identified to be driven by chronic inflammation, altered hormone and metabolic pathways and signalling, and changes in diet and lifestyle [25, 87]. These drivers result in disrupted microbial balance across multiple body sites, contributing to microbial imbalance and altered microbial composition and function (Fig. 4). These obesity-driven microbial alterations not only contribute to local and systemic dysbiosis, but are also increasingly recognised as potential influencers of reproductive health outcomes including infertility and pregnancy related complications [85].

### Vaginal Microbiome

Reproductive-aged females considered as overweight and/or obese show increased alpha diversity through microbes such as *Megasphaera* and *Mobiluncus* in combination with a reduced abundance of the genus *Lactobacillus* in the vagina [70, 87–89]. This shift has been postulated to be driven by reduced oestradiol production and subsequent impairment of glycogen synthesis in the vaginal epithelium. As a result, *Lactobacillus* abundance declines, leading to an increased pH, cytokine levels, and relative abundance of obligate anaerobes. Interestingly, weight loss has been shown to beneficially shift the vaginal microbiome towards a *Lactobacillus* dominated environment [70, 88].

At the species level, the vaginal microbiome in obese females has been found to be phenotypically altered, with lower levels of *Lactobacillus crispatus* and higher levels of *Lactobacillus iners*, *Dialister spp*., *Anaerococcus vaginalis*, and *Prevotella timonensis* [85, 87, 89]. Due to the depletion of lactic acid producing microbes—such as *Lactobacillus spp*.—in the vagina of women with obesity, the increased vaginal pH increase has been shown to increase the proliferation of the yeast genus *Candida* which also significantly alters the microbial composition [88].

Females with obesity have a higher incidence of BV characterised by an overgrowth of microbes such as *Gardnerella vaginalis*, *Fannyhessea vaginae*, *Megasphaera spp.*, *Prevotella spp.*, and *Sneathia spp.* accompanied by a simultaneous decline in *Lactobacillus spp.* [90]. Furthermore, several studies have theorised that alterations in the vaginal microbiome may influence adverse pregnancy outcomes through increased microbes associated with preterm birth—such as *F. vaginae* (formerly known as *Atopobium vaginae*)—identified more frequently and in increased abundances in obese pregnant women when compared to healthy weight pregnant females [70, 85, 87, 88]. However, further research is required to elucidate any link between obesity and adverse pregnancy outcomes due to microbial disturbances.

### Gut Microbiome

Unlike the vaginal microbiome, the gut microbiome has been identified to have decreased microbial diversity in overweight/obese females [25]. Harmful microbes present in the gut due to obesity can result in reduced gut barrier integrity and increased inflammation further increasing gut inflammation and increasing the risk for systemic inflammation across multiple body sites [91]. In addition to this, reduced microbial diversity, obese women also showed an increased abundance of Proteobacteria, Firmicutes, Fusobacteria, Mollicutes and *Lactobacillus spp*., a higher Firmicutes/Bacteroidetes ratio, and reduced *Bifidobacterium* [7, 25]. It has been hypothesised that changes to the gut microbiome are driven by an obesogenic diet rather than the increased weight itself [25, 46]. This may be due to a positive feedback loop where the altered gut microbiome produces specific metabolites affecting the gut-brain axis, potentially contributing to further weight gain [25]. An imbalance in the gut microbiome may also contribute to increased fat storage due to altered microbes having an increased efficiency in extraction of energy from food.

### Oral and Skin Microbiomes

Obesity has also been linked to oral microbiome dysbiosis, often accompanied by poor oral health that can further perpetuate microbial imbalance. In females with obesity, an increased in the abundance of the genera *Capnocytophaga* and *Streptococcus* was observed when compared with females at a healthy weight [92]. Additionally, increased levels of skin *Corynebacterium* has been found to significantly correlate with increasing levels of obesity wherein obesity has correlated with a significant influence of $\:\alpha\:$-diversity, $\:\beta\:$-diversity, and overall community composition when compared to healthy weight females [65, 93].

Overall, obesity has been identified as a driver of widespread microbial disturbances across multiple body sites, with consistent patterns of dysbiosis emerging across the vaginal, gut, oral, and skin microbiomes. These changes have important implications for reproductive health and disease risk, highlighting the need to include consideration of obesity as a key factor when examining female microbiome dynamics.

## Physical Activity

Physical exercise has also been shown to directly influence the composition of the vaginal, gut, and oral microbiomes, along with influencing the skin microbiome indirectly through factors such as physical contact, sweating, equipment usage, and other environmental exposures. Exercise has been identified to promote homeostasis in microbial sites through a reduced inflammatory response [28, 36].

### Vaginal Microbiome

The impact of physical exercise on females has been found to have two key impacts. Females that partake in regular physical exercise have been shown to have an increase in vaginal $\:\alpha\:$-diversity and a decreased risk of transitioning into a vaginal community profile associated with negative reproductive health outcomes (community state type (CST) IV) [28, 70].

### Gut Microbiome

Whilst the influence of non-dietary factors on the gut microbiome has to date been underexplored, lack of exercise has been shown to significantly impact large bowel health through increased risk of colorectal cancer in patients not undertaking regular exercise [25]. Additionally, individuals who undertook regular exercise showed an increase in the relative abundance in a range of microbial communities including *E. rectale*, *Akkermansia muciniphilia*, *Faecalibacterium prausnitzii*, *Eubacterium hallii*, and *Gordonibacter massiliensis* and genera *Roseburia*,* Lachnospuraceae*, and *Erysipelotrichaceae.* This increase in microbes resulting from exercise have been identified to be beneficial for SCFA production with immunomodulatory characteristics [94]. Overall, increased exercise has been associated with greater gut $\:\alpha\:$-diversity, as well as increased relative abundance of the phylum Firmicutes and the family *Clostridiales* [94, 95].

### Oral Microbiome

The oral microbiome has been identified to have an inverse relationship to the gut where increased physical activity has been associated with a decreased overall diversity, encompassing a decrease in both $\:\alpha\:$-diversity and $\:\beta\:$-diversity. This has been found to be associated with an increase in the relative abundance of Bacteroidetes and *Neisseriaceae* and a decrease in the abundance of Firmicutes, *Corynebacteriaceae*, and Flavobacteriia communities [95].

### Skin Microbiome

The relationship between physical activity and the skin microbiome remains unclear, with factors such as physical contact, sports equipment, and environmental exposures found to significantly influence skin microbial composition, making it difficult to isolate the direct effects of exercise alone. Physical activity has been associated with establishing and maintaining skin mechanics, immune defence, and skin microbiome liveliness. For skin microbiota, significant microbial alterations occur due to physical contact between athletes, promoting transmission of microorganisms [95, 96]. However, increasing contactless activities such as running and walking, has been shown to significantly increase the relative abundance of the fungi Tinea pedis due to the increase in body sites establishing moist, warm and sweaty environments in which the microorganism thrives [96].

Overall, physical activity influences microbial composition across multiple body sites. This promotes beneficial diversity in the gut and vaginal microbiomes while exerting more complex effects on the oral and skin microbiomes through both direct and indirect mechanisms.

## Stress

Psychological stress is another factor influencing microbial composition across female body sites due to the pro-inflammatory and cytokine response resulting from stressful events, or chronic stress. In females with high stress, reduced oestradiol, LH, luteal phase progesterone, and increased FSH are commonly identified compared to low stress females [97].

### Vaginal Microbiome

Stress has been identified to influence microbial composition in the vaginal microbiome via disruption of glycogen deposition homeostasis in the vagina with cortisol acting as a glycogen deposition inhibitor. Glycogen serves as the primary energy source for dominant *Lactobacillus* species in the vagina and when disrupted, *Lactobacillus* abundance declines, reducing its protective influence and increasing the likelihood of dysbiosis and vaginal infections such as BV and STIs [7, 70, 74, 98, 99]. High psychological stress has also been associated with increased disease severity and disease persistence for STIs, BV and other vaginal infections [100]. Additionally, in women presenting with high levels of stress and depressive symptoms, symptoms of vaginosis (vaginal itching/irritation, burning sensation during urination, fishy vaginal odour, mild discomfort) have been found to be more common, further supporting a potential relationship between stress and vaginal health [101]. In addition, increased stress has been identified to enrich dysbiotic vaginal environments and establish CST-IV through impairment of the hosts immune response, influencing other behavioural practises such as unprotected sexual intercourse, alcohol consumption, smoking and douching [99, 100].

### Gut Microbiome

Links between stress and the gut microbiome are becoming more established through the gut-brain axis that modulates gut microbiome communities [25]. Females with increased stress showed an increased abundance of *Escherichia*, *Shigella*, and *Blautia* in the gut. Like the vaginal microbiome, stress may alter behaviour and mood to influence eating habits and directly influence gut microbial composition [101]. For example, stress influences eating behaviours and a diet with low fibre and high sugar has been identified to influence the abundance of *Escherichia coli* in the gut resulting in the release of endotoxins to influence negative-moods furthering stress and a negative mood which can potentially act in a continuous cycle [101].

### Oral Microbiome

Psychological stress has also been demonstrated to link with oral microbiome abundance where increased stress correlated with reduced $\:\alpha\:$-diversity, *Prevotella*, *Neisseria*, *Streptococcus gordonii* and *Corynebacterium* through changes in hormone release (cortisol, adrenaline, noradrenaline, and mucin), altering microbial composition [102].

### Skin Microbiome

Current information regarding the impact of stress on the skin microbiome is limited, however, increased stress has been linked with decreased in $\:\alpha\:$-diversity on the skin resulting in an increased abundance of acidophilic and anaerobic microbes potentially linked to skin inflammation and overall dysbiosis [103]. Microbes such as *Corynebacterium* and *Streptococcus epidermis* have been suggested as opportunistic microbes on the skin with abundance increased during stressful events [26]. Additionally, some skin diseases have been referred to as stress-related, encompassing skin disease where stress exacerbates symptoms such as acne or eczema [26, 103].

Stress influences on microbial communities through both direct biological mechanisms and indirect behavioural changes, driving dysbiosis across multiple body sites. These widespread effects highlight the need to consider stress management as an integral component in strategies aimed at maintaining microbial balance and promoting female health.

## Hygiene Practices

Hygiene practices also play a key role in shaping microbial communities across the body through influence on the local environment, microbial diversity, and abundance. Behaviours such as personal cleaning, use of hygiene products, and contact with environmental surfaces can support healthy microbial balance or contribute to dysbiosis, depending on their frequency, method, and type of products used [2, 5, 22]. While specific impacts vary between body sites, hygiene has been shown to affect the vaginal, gut, oral, and skin microbiomes most directly, altering microbial composition through changes in pH, nutrient availability, and local inflammatory responses. Understanding these interactions supports the importance of targeted, moderate hygiene practices to maintain ideal microbial health.

### Vaginal Microbiome

Hygiene practices such as vaginal douching and washing have been identified to be key contributors to vaginal dysbiosis and increased BV, STIs and HIV infections due to a reduction in *Lactobacillus spp.* [5, 7, 76, 104]. The use of any type of vaginal cleaning product—such as sanitary napkins, douching products, moisturisers, lubricants, and vaginal washes—has been identified to promote a proinflammatory environment that can promote vaginal epithelial cell death, considerably altering microbial composition and weakening the vaginal barrier [5, 7, 23, 105]. Females who utilise cloths or sanitary napkins during menses have been identified to be more likely to present with vaginal dysbiosis (CST-IV) rather than a healthy environment (CST I) [36, 106].

Females who participate in vaginal douching have also been found to be 1.2–1.5 times more likely to develop BV than non-douching females depending on douching frequency, with females using any feminine hygiene product identified to be 3 times more likely to report adverse vaginal health outcomes such as BV, STIs or UTIs [70, 107]. Interestingly, females that participated in vaginal douching showed a reduced abundance of *Lactobacillus*—specifically *L. crispatus*— and an increase in *Mageeibacillus indolicus*, *F. vaginae*, *Leptotrichia spp*., *Megasphaera spp*. and *G. vaginalis* communities [23, 76, 108]. The process of douching alters the vaginal microbiome so significantly, that cessation alone has been identified insufficient to restore the microbial balance with additional interventions required to restore the vaginal microbiome [109].

### Gut Microbiome

Hygiene practices such as kitchen cleanliness and cross-contamination awareness are widely recognised for their role in influencing gut microbial composition—mainly through foodborne illnesses from microbial colonisation of pathogenic microbes such as *E. coli*, *Campylobacter jejuni*, *Salmonella Chester*, *Salmonella Enteritidis*, and *Salmonella Berta*. While hygiene practises have scarcely been identified to significantly alter the gut microbiome in a similar way to vaginal douching, oral hygiene, or skin hygiene impact their retrospective microbiomes [110]. However, exposure to household animals has been linked to increased taxonomic diversity in the gut and increased *E. coli* isolates in pregnant females than non-animal counterparts [111]. Additionally, water sanitation and infrastructure levels have been directly linked with gut microbial alterations. Reduced water sanitation and infrastructure has been linked with a lower gut microbiome diversity and reduced abundance of *E. coli* [111]. Low-cleanliness environments have also been associated with an increased gut microbial diversity. Increased sanitation levels and decreased environmental microbial exposures associated with the Western lifestyle have also been linked to reduced gut diversity [112]. Increased urbanisation has significantly been linked with decreased microbial diversity due to decreased environmental microbial exposure [113].

### Oral Microbiome

Poor oral hygiene has been found to result in ecological shifts and subsequent or microbial dysbiosis and which results in the accumulation of carcinogens and an increased inflammatory state [22, 114, 115]. A lack of oral hygiene has also been found to promote the accumulation of microbiomes within biofilms in the oral cavities, creating a pathogenic environment and disease [115]. An increase in brushing frequency has been shown to reduce *Streptococcus* abundance with poor oral hygiene associated with an increase in several microbes associated with microbial dysbiosis such as *Prevotella denticola*, *Leptotrichia spp*., *Saccharibacteria* spp., *Lachnospiraceae spp*., *Tannerella spp*., *Fusobacterium nucleatum* [116]. As such, poor oral hygiene has generally been found to be associated with a higher $\:\alpha\:$-diversity independent of brushing frequency including a significantly increased abundance of *Staphylococcus spp.* associated with oral disease [117].

### Skin Microbiome

Different hygiene habits have been found to have a profound influence on the skin microbiome with hygiene the largest modifiable factor for skin microbial diversity [2]. Modifications in hygiene practices alter the skin microbiome and metabolome with the interaction dependent on the location and product used [118]. Hygiene practices enable good health, however cleaning can also remove lipids and moisture from the skin which resulting in skin irritation and microbiome dysbiosis [119]. Products such as moisturisers, cleansers, and soaps can all promote the growth of opportunistic microbes by providing a nutrient source [120]. Moisturisers promote the growth of microbes such as *Staphylococcus* and *Cutibacterium*. Certain hygiene habits, such as infrequent or excessive bathing, can decrease the overall bacterial diversity of the skin reducing the abundance of microbes such as *S. epidermis*, *Lactobacillus spp.*, *Burkholderis spp*., *C. acnes* and increases *Staphylococcus*,* Corynebacterium*,* Cutibacterium*,* and Micrococcus* [2]. Excessive use of cosmetics can also significantly reduce $\:\alpha\:$-diversity dependent upon the chemical content. However, when cosmetics are used regularly or over a long period of time, cosmetic products have been suggested to contribute to the diversification of the skin microbiome. Additionally, contact with animals has a significant impact on the skin microbiome with those living with animals sharing similar microbes to their pets than those living without animals suggesting microbial exchange between pet and owner [2].

In summary, hygiene practices can support or disrupt microbial balance, with poor or excessive practices key contributors to dysbiosis in the vagina, oral cavity, and skin. This data emphasises the importance of targeted, moderate hygiene interventions to preserve healthy microbial communities.

## Reproductive Health

### Sexual Activity

#### Vaginal Microbiome

Sexual activity and practices play an important role in shaping the human microbiome, particularly within the vaginal environment. Behaviours such as frequency of sexual activity, number of partners, and protective measures can influence microbial balance, sometimes increasing susceptibility to infections and dysbiosis [121, 122]. While most research to date has focused on the vaginal microbiome, emerging evidence suggests that sexual activity also impacts the gut, oral, and skin microbiomes through shared environments, partner microbiome exchange, and increased risk of pathogen transmission [123–125]. These interactions provide insight into how sexual behaviours may affect overall microbial health and provides opportunities for prevention and intervention strategies.

Sexual activity and practises significantly influence the vaginal microbiome through changes in microbial abundances and increase infection susceptibility. Sexual activity—and more specifically unprotected sexual activity and increased sexual partners— has been found to result in a loss of *Lactobacillus* in the vagina causing a shift to more pathogenic community types (CST-III and CST-IV), increasing the risk of developing BV [122, 126]. In addition, increased frequency of penile-vaginal sex and unprotected sex results in an enrichment of *Gardnerella vaginalis*, *Sneathia* and BVAB due to alterations in vaginal pH [7, 36, 126]. Sexual practises such as the use of lubricants, use of sexual accessories, and vaginal intercourse after anal or oral intercourse have been found to be associated with a high-risk environment and increased incidence of BV, STIs, along with significant changes in the vaginal microbiome [5, 121]. In heterosexual couples, uncircumcised males increase the prevalence of male to female transfer of BV, also increasing proportionally to new partners [121]. Increased BV risk has also been significantly correlated with women who have sex with women (WSW), in particular, new sexual partners [23, 76, 127]. Reducing unprotected sexual encounters from multiple male or female partners has been suggested as a strategy to reduce the risk of BV incidence and recurrence [128].

#### Gut Microbiome

Whilst current research on the influence of sexual practices and hygiene on the gut, oral, and skin microbiomes are lacking, there is some information regarding the microbial change resulting from sexual practices. There is little to no information regarding the influence of sexual practise on the female gut microbiome, with studies focussed on male gut microbial changes in men who have sex with men (MSM) due to increased risk of HIV infection. Whilst sexual practises have been shown to influence all microbiomes, specific microbial shifts or diversity analyses remain scarce. Couples in close relationships have been demonstrated to contain a higher diversity and overall richness in their microbial composition when compared with individuals living alone with spouses showing more similar gut microbiota composition than siblings indicating significant interactions occur between microbiomes [123].

#### Oral Microbiome

Increased sexual partners along with an overall higher frequency of oral sex may be associated with an increased risk of infection by viruses such as *Epstein-Barr Virus (EBV)* and overall transmission of pathogens due to microbial dysregulation and dysbiosis [124, 129]. However, oral sex is considered a low risk activity due to various protectors such as physical barriers, oral hygiene, ethnicity, and overall health [129].

#### Skin Microbiome

The lack of investigations into the skin microbiome in relation to sexual practices suggests some impact on resident microbes, however, similarly to the gut, couples in close relationships have been identified to share similar microbiota from constant contact. In addition, factors such as hygiene practices, skin care products, pet ownership, diet and many others influence the skin microbiome significantly more [125].

In summary, sexual activity drives shifts in the vaginal microbiome that can result in increased susceptibility to BV, STIs, and other infections. These connections highlight the potential for targeted interventions—such as safer sexual practices—to preserve microbial balance and promote reproductive health.

### Contraceptives

Contraceptives have also been identified to influence microbial composition across different body sites through their effects on hormones and other physiological processes. Hormonal contraceptives can be taken orally, inserted vaginally, implanted under the skin, and injected. Contraceptive types, including hormonal methods containing estrogen or progestin, along with non-hormonal options such as barrier methods or copper IUDs, may create conditions supportive or disruptive to microbial community balance [23, 28]. These shifts can vary depending on factors such as the hormonal composition, method of delivery, and duration of use, and can lead to changes in microbial diversity and abundance across the vaginal, gut, oral, and skin microbiomes.

#### Vaginal Microbiome

The impact of female contraceptives on female reproductive health is an area of intense debate, with a range of differing opinions specific to each method. Female contraception methods include hormonal contraceptives containing estrogen, progestin, or both, administered via pills, injectables, implants or intrauterine devices (IUDs). Other methods include non-hormonal or barrier methods such as condom use and cervical caps [23]. Estrogen-based hormonal contraceptives, such as pills and vaginal rings have been found to be associated with significant reduction in BVAB including *G. vaginalis* and *F. vaginae*, along with simultaneously promotion of the colonisation of the protective *Lactobacillus spp.* [5, 130]. In contrast, progestin-only contraceptives have been shown to reduce *Lactobacillus* abundance in the vagina and to repress epithelium proliferation [7, 28]. Although the exact mechanisms driving these microbial shifts remain unclear, some evidence has linked progesterone-based and combined contraceptives with decreased BV risk [28, 98]. Overall, hormonal contraceptives have been broadly correlated with a reduced risk of vaginal dysbiosis and decreased susceptibility to BV and STIs due to oestrogenic stability [23, 76, 130, 131].

Nevertheless, several studies have reported a link between hormone contraceptive pills and increased susceptibility to gynaecological disorders from genital inflammation [131]. The use of copper IUDs has been correlated with an increased vaginal colonisation of BVAB such as *G. vaginalis* and *F. vaginae* along with an increased prevalence of BV [23, 127, 132, 133]. These microbial changes potentially result from disruption of the vaginal microbiome during the insertion process rather than the type and activity of the hormonal contraceptive itself [70]. Factors such as hormonal composition, release method, and the presence or absence of a hormonal free period have all been found to influence the vaginal microbiome [28]. As an example, women with a history of contraceptive use consistently exhibited a greater likelihood of having a *L. crispatus* dominated vaginal microbiome than non-users. Another example includes the increased abundance of optimal vaginal microbes such as *L. crispatus with* the use of barrier contraceptive methods [5, 134].

#### Gut Microbiome

The influence of contraceptives on the gut microbiome contributed to be widely debated, with several studies reporting compositional changes, with others reporting no significant compositional changes. The use of oral combined hormone contraceptives (CHC) has also been associated with slight reductions in $\:\alpha\:$-diversity, however, no significant difference in $\:\beta\:$-diversity was observed in the gut microbiota of CHC users and non-CHC users [135]. To date, the use of oral contraceptives has not correlated with significant changes in microbial communities as naturally occurring hormones more significantly influence the composition of gut microbiome [136]. In a study by Brito et al. significant changes in $\:\beta\:$-diversity between the control group and contraceptive group were reported, however, statistical significance was lost once the data was adjusted for confounders such as age, BMI, and physical activity status suggesting the need for more investigations [137].

#### Oral Microbiome

Contraceptive use has not been found to be associated with microbial changes in the oral microbiome of reproductive-aged females [21, 138]. A systematic review investigated the influence of oral contraceptives on oral microbiome composition identified oral contraceptives may increase susceptibility to colonisation by *Candida albicans* and *Candida krusei*, and concluded that hormonal contraceptives were not associated with significant changes in the oral microbiome [139].

#### Skin Microbiome

Whilst the influence of contraceptives on the gut and oral microbiomes remains an area of active debate, significant associations between contraception and the skin microbiome have been observed. In particular, in relation to acne management and treatment. Oral contraceptives have been linked to decreased oil production on the skin from sebaceous glands, resulting in altered microbial abundance particularly from microbes that utilise the naturally produced oil or sebum as a primary food source [140]. Microbes such as *Corynebacterium kefirrestidentii* and *Cutibacterium acnes* have been identified to utilise sebum as a nutrient source, with the released free fatty acids contributing to acne formation. In contrast, *S. aureus* has been identified to be more abundant in conditions of low sebum levels [141].

Overall, contraceptive use, particularly hormonal methods, influences microbial composition primarily in the vagina and skin, to promote protective *Lactobacillus* colonisation and improve acne outcomes. However, the effects on the microbiomes of the gut and oral cavities remain less clear and warrant further investigation.

## Conclusion

This review has highlighted the substantial influence of modifiable factors on the composition and function of the female microbiome across the vagina, gut, oral cavity, and skin. Individual lifestyle factors such as diet, alcohol use, smoking, and obesity emerged as the most dominant drivers of microbiome composition, with physical activity and stress showing more moderate effects, and sexual activity specifically impacting the vaginal microbiome. Across the anatomical sites identified, these factors have been found to primarily act through the modulation of cytokines and chemokines, modulation of female hormonal profiles, triggering inflammation that can disrupt microbial balance and drive adverse health outcomes. While individual associations have been identified, the combined and site-specific interactions of these behavioural factors remain poorly understood. Advancing this field will require integrated, multi-site microbiome studies that incorporate a wider range of modifiable variables and unmodifiable variables (such as hormones and pharmaceutical aspects), to enable the development of targeted interventions focused on the microbiome to improve female health across their lifespan.

## Data Availability

No datasets were generated or analysed during the current study.
